# Qualia Could Arise from Information Processing in Local Cortical Networks

**DOI:** 10.3389/fpsyg.2013.00121

**Published:** 2013-03-14

**Authors:** Roger Orpwood

**Affiliations:** ^1^Centre for Pain Research, Department for Health, University of BathBath, UK

**Keywords:** re-entrant feedback, qualia, information processing, local cortical network, network feedback, oscillatory activity

## Abstract

Re-entrant feedback, either within sensory cortex or arising from prefrontal areas, has been strongly linked to the emergence of consciousness, both in theoretical and experimental work. This idea, together with evidence for local micro-consciousness, suggests the generation of qualia could in some way result from local network activity under re-entrant activation. This paper explores the possibility by examining the processing of information by local cortical networks. It highlights the difference between the information structure (how the information is physically embodied), and the information message (what the information is about). It focuses on the network’s ability to recognize information structures amongst its inputs under conditions of extensive local feedback, and to then assign information messages to those structures. It is shown that if the re-entrant feedback enables the network to achieve an attractor state, then the message assigned in any given pass of information through the network is a representation of the message assigned in the previous pass-through of information. Based on this ability the paper argues that as information is repeatedly cycled through the network, the information message that is assigned evolves from a recognition of what the input structure is, to what it is like, to how it appears, to how it seems. It could enable individual networks to be the site of qualia generation. The paper goes on to show networks in cortical layers 2/3 and 5a have the connectivity required for the behavior proposed, and reviews some evidence for a link between such local cortical cyclic activity and conscious percepts. It concludes with some predictions based on the theory discussed.

## Introduction

An important development in work exploring mechanisms underlying consciousness has been a move from the important search for Neural Correlates of Consciousness (Rees et al., 2002; Koch, 2004) to a deeper look at mechanisms involved, a search for Explanatory Correlates of Consciousness (Seth, 2009), the neural processes that can actually account for fundamental properties of conscious experience. A key element in this work is the search for mechanisms that can account for the phenomenal aspects of the contents of consciousness, such as perceptual experiences.

An important set of ideas about the locus of conscious percepts revolves around the concept of re-entrant feedback. Originally proposed by Edelman (1992, 2003) it has been explored by a number of others (e.g., Lamme and Roelfsema, 2000; Bullier, 2001; Pascual-Leone and Walsh, 2001). The basic idea is that information entering the cortex is initially fed-forward through the hierarchy of areas in the sensory cortex, onto motor cortex, and prefrontal areas, but that concluding network states in higher regions would be fedback to modulate and integrate the incoming sensory stream. It has been seen to provide the basis for perceptual organization (Lamme and Spekreijse, 2000) where higher level conclusions about the meaning of sensory information are used to fine-tune the lower level building blocks until an agreement is achieved, and has parallels with modeling approaches such as Grossberg’s Adaptive Resonance Theory (Grossberg, 1976) and his ideas about the fine tuning of sensory inputs via top-down feedback (Raizada and Grossberg, 2003). There is growing experimental evidence that this re-entrant feedback is crucial to the development of conscious percepts (e.g., Haynes et al., 2005; Silvanto et al., 2005; Boehler et al., 2008).

The feedback can be at different levels. For full-blown conscious visual perception Lamme has argued that the re-entrant activity arises from the prefrontal cortex as part of attentional activation following an initial feed-forward stream from the visual cortex (Lamme, 2010). Rees has also argued for the need for this higher level re-entrant activity to be a key feature of conscious vision (Rees et al., 2002; Rees, 2007). It would seem to be an essential component of access consciousness, that is the phenomenon whereby information in our minds is accessible for verbal report, reasoning, and the control of behavior. Block (1998) distinguished access consciousness from the phenomenal experiences of qualia. Lamme (2010) has gone on to argue that re-entrant activity within the visual cortex itself could lead to conscious percepts during iconic memory. Boehler et al. (2008) showed that such local re-entrant feedback can lead to early awareness of sensory information which was far too rapid to involve prefrontal attentional feedback.

**Figure 1:**
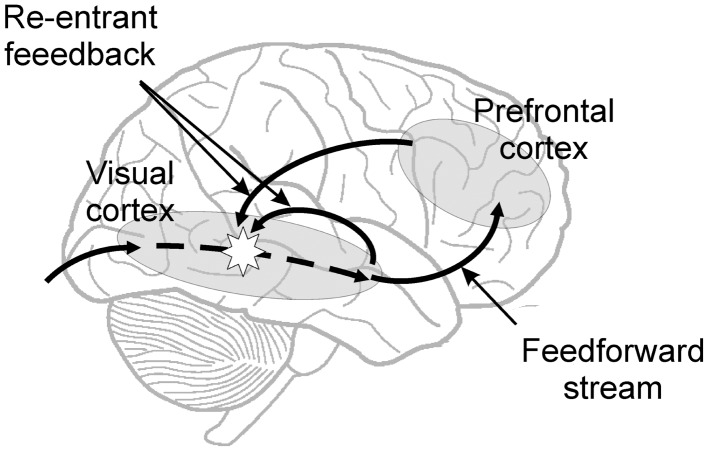
**Diagram showing the feed-forward sweep and re-entrant feedback of information felt to be necessary for conscious percepts**. The conscious percept itself could arise in some way from local activity in the sensory cortex stimulated by the re-entrant feedback, as indicated by the star.

In a completely different approach Zeki and colleagues showed that conscious perception of color is perceived before motion (Moutoussis and Zeki, 1997; Zeki and Bartels, 1998), and therefore argued for the existence of micro-consciousness within given areas of the cerebral cortex (Zeki, 2001). Taken together the ideas of re-entrant feedback and micro-consciousness seem to be arguing that re-entrant feedback activation is necessary for conscious percepts, but that the site of the neural activity from which they emerge could be quite a local one (Figure 1).

This line of argument seems to be moving toward a conclusion about a set of neural activities that lead to the generation of consciousness. The unanswered question still of course is that, even if these mechanisms can be shown to lead to conscious percepts, what is it about the local activity that generates experience? How can this defined neural activity lead to phenomenology? What is the process whereby qualia are formed? It is the essential question behind the hard problem of Chalmers (1995) or the explanatory gap of Levine (1983). The thinking around re-entrant feedback appears to suggest that there is something about the activity of localized areas of cortical tissue that under top-down feedback activation transforms their activity in some way that leads to an experiential outcome. This paper explores the behavior of local cortical networks under the activation of top-down feedback from an information processing perspective, to see if there is anything in that behavior that could lead to the emergence of qualia.

Tononi proposed that conscious states are characterized by the fact that they involve information that is highly integrated and highly differentiated, and went on to propose measures to quantify the informational relationships generated by networks (Tononi, 2004; Balduzzi and Tononi, 2009). This paper also takes an information-based perspective to explore the pattern recognition capabilities of networks of pyramidal cells. It analyses the behavior of networks encouraged by top-down feedback to engage in extensive local feedback to themselves and to establish attractor states, and to examine how they process the fedback representations they generate as outputs. It is argued that the ability to repeatedly recognize their own representations in cycles of local feedback could lead to the generation of qualia. It develops further previous studies on self-reflective neural circuits (Orpwood, 2007, 2010).

## Information Processing in Local Networks

### Spatial pattern recognition

**Figure 2:**
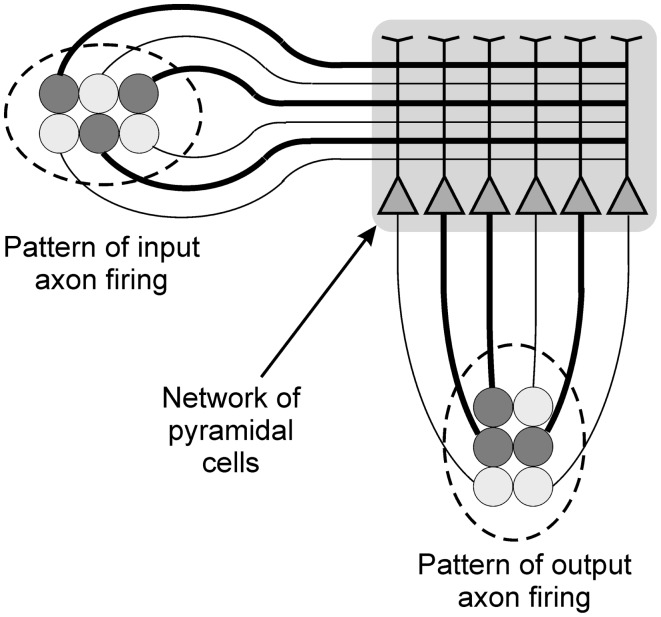
**Diagram illustrating the processing of spatial patterns of information by a network of pyramidal cells**. The darker components of the input and output patterns, and the darker axonal connections, both represent firing activity.

The paper focuses on the behavior of networks of pyramidal neurons exposed to spatial patterns of information. For individual pyramidal cells these spatial patterns would consist of the distribution of inputs over the surface of the cell during any given epoch of time in the order of a few milliseconds. For networks spatial patterns would again be the distribution of inputs to the various cells within the network (see Figure 2). Much of the discussion of pattern recognition in neurons relates to temporal patterns, constituting a form of neural “code” (Theunissen and Miller, 1995; Rolls and Treves, 2011). This paper argues that although temporal patterns clearly are processed, both individual pyramidal cells and networks of those cells are also very able to recognize spatial patterns, and that this process is particularly important to the process of perception, and to the argument below for the emergence of qualia. The paper therefore reflects the views of Harris that the fundamental currency of information processing in the cortex is the spatial pattern of firing activity of single assemblies of neurons (Harris, 2005).

Modeling work has shown that individual pyramidal cells should have the ability to recognize spatial patterns in the input information that they receive (Mel, 1992; Orpwood, 1994). Receptor sensitivities become adjusted as a result of training. Following this learning the cumulative excitatory post-synaptic potential (EPSP) generated in the soma reflects the degree to which the current input pattern is similar to ones experienced during training. If the depolarization reaches threshold then the cell essentially recognizes the input pattern, and can signal that recognition by firing. Many different input patterns can be learned, but the only output available to the cell is to signify that it has recognized the input. Of course rates of firing may reflect depth of depolarization, and cells can also demonstrate different types of firing response, such as regular or burst firing, but they cannot provide information about the identity of their input patterns. So a cortical pyramidal cell can recognize spatial patterns and can signal recognition but cannot differentiate between the different input patterns that it has learnt. This inability is overcome when cells are connected in networks, and where the input pattern is fed to the cells within this network. If the input pattern is sufficiently similar to those experienced during training, then across the network a number of different cells will fire in response to the input pattern. The firing indicates that the input pattern has been recognized, and the distribution of this activity is a unique response of that network to the input pattern. It constitutes the network’s output, and it provides information that reflects the nature of the recognition that has taken place, the identity of the input pattern to that network (Figure 2).

### Analysis of information processing

**Figure 3:**
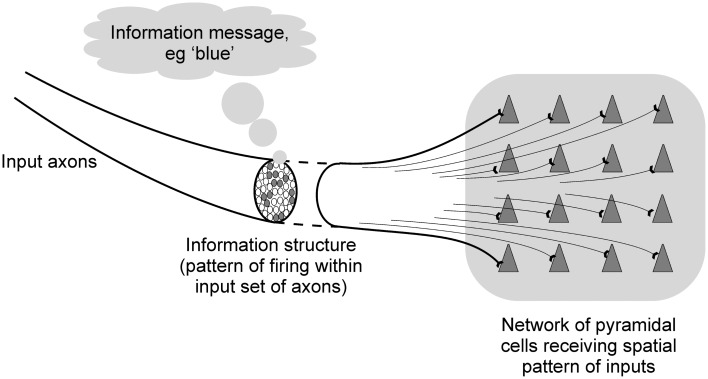
**Diagram illustrating the difference between information structures and information messages in cortical information processing**.

This paper aims to examine the behavior of such networks from an information processing point of view. The term “information” can be used to mean quite different things. In terms of cortical information processing it is important to distinguish between the information message, and the information structure. The information message is what the information is about, what it is communicating. The information structure is the physical form by which the information is carried, the way in which the information message is embodied. For example, firing patterns being communicated to the V4 area of the visual cortex may include information structures that relate to the color blue in the visual environment. The information message is the color blue, the information structure is the firing activity that represents the blue color (see Figure 3).

The information structure is a physical entity and can be analyzed using the mathematical tools of information science. The information message is a more abstract concept and is at the core of phenomenology. The content of consciousness comprises information messages, not their structure. The whole mental world of individuals is made up of information messages. On the other hand the neurobiological mechanisms taking place in the brain are engaged with processing information structures. The challenge to neurobiology is to explain how the brain is able to release into awareness the information messages that are embodied in the information structures of physical neural activity.

The relationship between the information structure and the information message is a crucial one. Consider initially a basic information sender. The sender is configured to initiate the generation of an information structure following some internal change of state. For example, the sender might be a simple timer. The timer has some form of internal clock, and it is configured to send out a signal when the clock reaches a certain time. The signal is the sender’s output information structure. For that sender it represents the event “time reached.” There is of course no phenomenology involved. The sender does not experience the event. It simply reacts to the event by generating an information structure. For that sender “time reached” is the information message. The signal that it generates is the information structure. The information structure represents the information message, but only for that sender.

For a receiver of this information the information structure can represent an infinite number of different messages. Consider an information receiver that has the capability to recognize the information it receives. If it is able to recognize an input structure then there will be some reaction to the presence of the input information it receives. It may just react by some activity that represents a level of familiarity, like the level of depolarization of a pyramidal cell. Or it may react by generating some specific activity which represents a recognition, like a pyramidal cell firing. Or it may react with a more complex activity like a network firing pattern, generating an information structure in its own right. But whatever it does in reaction to the input information it can only recognize the information structure. It cannot recognize information messages. But this doesn’t mean that the receiver cannot associate a message with the input structure. Consider a receiver like a network of pyramidal cells that will react to a recognition by generating a repeatable pattern of firing. If the network responds by always generating a particular information structure whenever it receives a certain input, then it is not just recognizing the input structure, it is identifying it. The firing response is its output information structure, and it is that network’s representation of the identity of the input structure. It does not of course know about the input, in the sense expressed by Clark and Karmiloff-Smith (1993) of the network’s knowledge being knowledge TO the network. It is not identifying the input in any experiential sense, but it is undertaking an identification. It is responding to an input by generating a repeatable information structure, and that structure represents the identity of the input to the network. If in this way the input information has some identity to the receiver then this identity constitutes an information message. The receiver has recognized the input information structure and generated an output information structure that represents the identity of the structure to the network, and thereby associates an information message with the input it has received.

**Figure 4:**
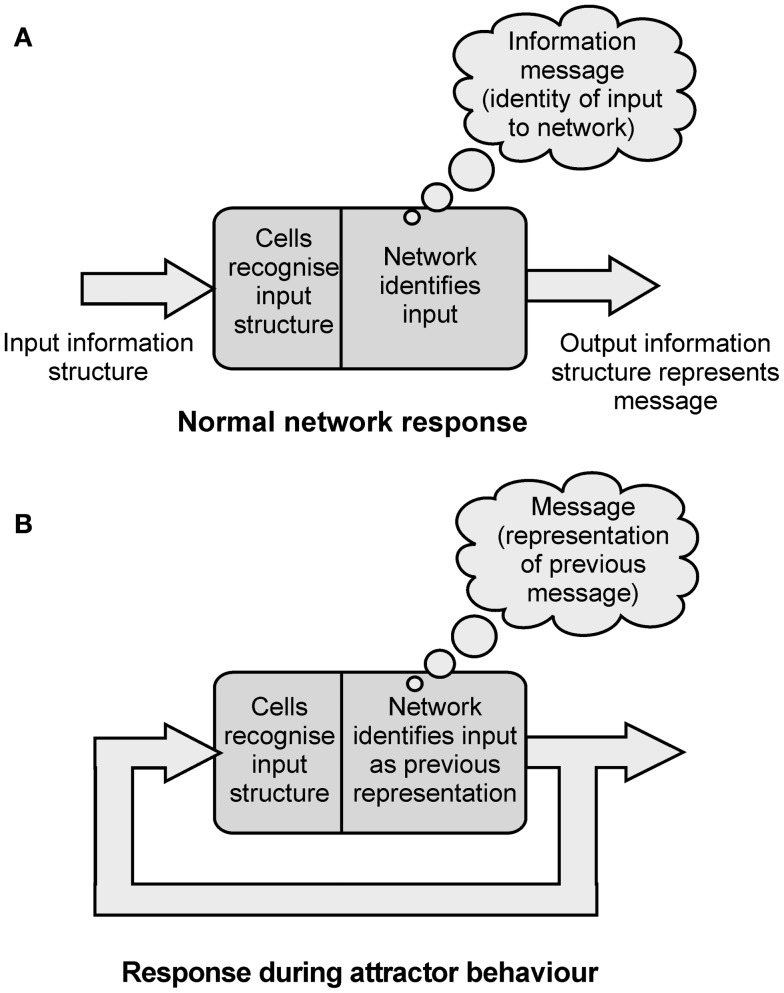
**(A)** Diagram illustrating the way a network can recognize information structures, assign messages to them, and generate output information structures that represent the message assigned. **(B)** Diagram showing how in attractor behavior the network assigns a message of “representation of previous message” to it’s feedback inputs.

So cortical networks are receivers of information, and can recognize and identify incoming information structures. The network’s cells cannot recognize a message in the incoming information, only its structure. Cortical networks can also act as senders of information. If an input information structure is identified, the network will respond by generating its own output information structure. The output structure represents the identity of the input structure to the network, the message that is assigned (Figure 4A).

### Impact of fed-forward and fed-back information

The network output can be communicated to a new downstream network. The new network can recognize the input structure it is receiving but not its message. The new input is just an information structure that the new network can recognize or not, depending on its learning. Similarly the network can feed its output back to its input again. This is local feedback, with the network’s cells feeding back directly to the cells in the network. The output from the first pass-through of information becomes the input for the next pass-through. Again if the fedback information is recognized, it is just the information structure that is recognized, not the information message.

So the network is able to recognize its initial input structure, identify a message, and generate an output that represents that message. If that output structure is locally fedback and recognized then the network is recognizing the representation it has just generated. But it cannot recognize messages. It cannot identify these fedback structures as its own representations. As far as the network’s cells are concerned they are just receiving input structures, and can respond to those structures by indicating a recognition or not.

The information processing becomes more interesting when the feedback activity has enabled the network to settle into an attractor state. Once an attractor has been established then the network will generate an output structure that is the same as its input structure (Amit, 1989). It is therefore self-recognizing. It is recognizing its own output structure, and generating an output in response that is the same. So what is the identity of the fedback input to the network? The output is a structure that represents the identity of the input to the network. But in an attractor state the input is the same as the output. So in this state the output is a representation of its own identity to the network. This is an important property of how a network responds when it is in an attractor state. Once an attractor state has been established, the output is a representation of its own identity to the network. But the cyclic feedback can continue, and if the attractor state is maintained for a few cycles then the identity will evolve. With each cycle the output is still a representation of the identity of itself, but each time this occurs the identity of itself is the identity of the previous output. With each cycle the output becomes a representation of the identity it had on the previous cycle.

As was argued above, the identification process assigns an information message to the incoming information structure. So in an attractor state the fedback information structure is identified as the network’s own representation of the last message assigned. This identity becomes the new assigned message. Each time an information structure is cycled through the network in an attractor state the message assigned is “representation of the previous message assigned.” As information is cycled through the network the information structure stays the same, and the information message also stays the same. The information message assigned on each pass is “representation of the message assigned in the previous pass” (Figure 4B).

This behavior is a unique response of networks that can recognize and identify information structures, and feed representations of the identity back to themselves. If they can be encouraged to develop attractor states, then the networks can demonstrate a unique behavior. They can repeatedly identify their own representations. This behavior, derived from a consideration of information processing in cortical networks, can be argued to provide those networks with experiential abilities.

### Implications of analysis for visual cortical processing

This section explores the implications of the previous analysis on the capabilities of networks undergoing attractor behavior. Consider a network in the V4 visual area that has undergone learning during the early development of the brain. Assume that as a result of this learning this particular network is now able to respond to the color blue in its visual environment. If attention is focused on the network then it will receive re-entrant feedback activation. This activation could enable the network to develop attractor behavior, as it repeatedly feeds back its own output back to itself. What is the nature of this network’s response to the patterns of input it receives? To the receiving network in V4 the sensory input pattern from the environment is just information with a particular information structure. The first time the network recognizes that information structure then at the point of recognition it will assign its own message to it, and generate an output with a structure that represents that internal message.

The network is undergoing local cyclic feedback. It is also under re-entrant activation, and this activation has enabled the network’s local feedback activity to settle into an attractor state. In this state the network recognizes its fedback information structures as representations of the previous internal messages assigned. To the network, each time its output is fedback it is identified in the same way. It is identified as its own representation of the previous message (Figure 4B).

So at the start of the cyclic activity, if the input information structure can be labeled as “x,” then the network recognizes the input structure and generates an output that represents the identity of the input, the identity of “x.” The identity of the input is simply what “x” is to the network. When this is fedback in an attractor state the input is identified, as argued above, as its own representation of the previous identity. So it is identified as its representation of the identity of “x.” The network is no longer just recognizing the pattern “x” as it did in the first cycle, it is recognizing a pattern that is this network’s representation of “x.” It is recognizing something about the way that the network embodied the identity of “x,” the way that it depicted it. There is a relationship between the identity and the representation of the identity. The identity is, in some way, embodied in the representational structure that the network generates. When it generates that representational structure the network has to portray what the identity is like to it. If the network then recognizes its own portrayal, it is recognizing what the identity was like to the network when it generated the representation. So the fedback representation is recognized as being what the identity is like to the network. Again there is no experiencing of “what it is like.” It is simply the relationship between the information and the network. Therefore, following the first feedback, the network is not recognizing what “x” is, but what “x” is like.

The output pattern is again fedback to the network, and identified as a representation of its previous output. So the identity of the feedback is “representation of what ‘x’ is like.” The network recognizes the new fedback input as a representation of what “x” is like to it. The representation that is recognized is again a depiction, a non-verbal description, but this time it is a depiction of what “x” is like to the network. It is not just recognizing the fact of what “x” is like, but how the network has depicted that likeness to itself. It recognizes it as a kind of abstract inner appearance, not just in a visual sense, but an appearance involving all of its attributes. So this time around the network is recognizing the input as how “x” appears to the network.

**Figure 5:**
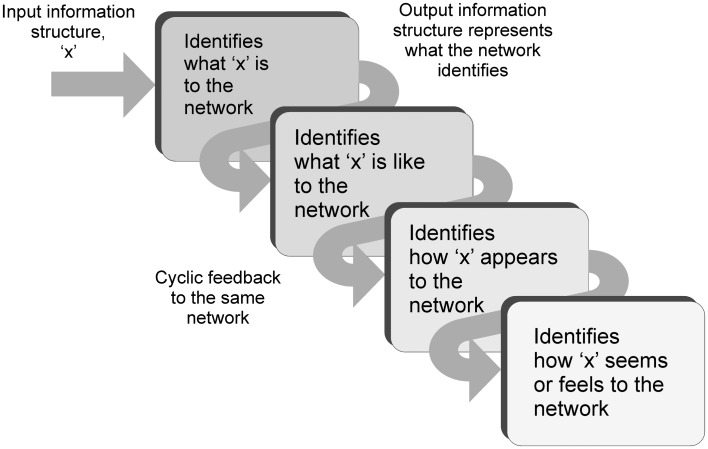
**Diagram illustrating the cycling of information through the same network during attractor behavior, and how the message assigned progressively changes**. The inputs are initially recognized as what the input is to the network, but the recognition changes on each pass to become what it is like, to how it appears, to how it seems.

The process continues and the feedback is then identified as “representation of how ‘x’ appears.” The network recognizes the input as a depiction of how “x” appears to it. It is a depiction of an abstract inner appearance. It is recognizing how its own internal appearance of “x” is depicted, its own inner abstract construct of “x,” its own inner image of “x.” What is recognized is how “x” seems to the network. Up to this point in the argument there has been no assumption of any experiential outcomes from the information processing taking place. However the conclusion after a few cycles of feedback is that the network is recognizing its input structure as how its original input “seems” to it. This outcome is identical to the concept of qualia. It is how the network experiences its input. To an observer monitoring the behavior of the network in V4 as the blue information is received, the initial network response is one of recognizing blue. With the cyclic feedback the response is observed to evolve to recognizing what blue is like to the network, to how it appears, to how it seems. To an observer the network is experiencing the blueness of blue. The blueness label can only be applied by the observer of course. To the network it has simply recognized how its original input information structure seems to it, how it experiences it (Figure 5).

Therefore the possible chain of events during sensory processing could be as follows. Information is received into a cortical sensory hierarchy and follows a fast feed-forward sweep through to motor and prefrontal areas. Attentional re-entrant feedback interacts with the sensory areas (equivalent to Lamme Stage 4 visual processing Lamme, 2010). The re-entrant feedback encourages networks within the sensory hierarchy to engage in local cyclic feedback and establish attractor states, depending on the focus of attention. Information is now locally fedback through these networks in a cyclic manner. On each pass-through of information the networks recognize their input information as a representation of the identity established in the previous pass. If the network initially recognizes an input, it simply recognizes its identity, what it is. If it recognizes a representation of its identity, it recognizes what it is like. If it recognizes a representation of what it is like, it recognizes how it appears. If it recognizes a representation of an appearance, it recognizes how it seems. In this way the top-down activation could enable the information received by local cortical networks to be experienced.

### Relation to other hypotheses

Most hypotheses concerning consciousness concern access to consciousness rather than the cause of the generation of phenomenal experience. There are quite a number of process models of consciousness that have been proposed, where there are executive processes going on whereby certain mental representations dominate over others to enable consciousness to emerge. Baars’s (1988) Global Workspace model is an important example of this approach. Distributed unconscious processors compete for access to a global workspace which controls and coordinates their activities. Those that are successful have their messages elevated to consciousness and broadcast throughout the cortex. The nature of the carriers of explicit representations is less important than what the system does with them. Such theories have been very successful in guiding thinking about the processes that are necessary for consciousness to emerge. However they have nothing to say about the mechanism of generation of phenomenal experience. Others have focused more closely on relating phenomenal experience with the vehicles of explicit representation in the cortex. For example O’Brien and Opie (1999) developed a theory which linked connectionism with experience. They saw consciousness as a myriad of individual phenomenal experiences, where each experience correlated with a pattern of stable activity in a localized region of a brain-wide connectionist network. However they did not attempt to show a causal link between the pattern of stable activity and the emergence of phenomenal experience. The current paper takes a similar view that local settled activity correlates with experience, but the stable activity is that of stable attractor states, and the stability is required to enable information structures to be cycled through the network to allow higher-order representations to emerge. The key point of the paper is a detailed argument for how the nature and operation of these states can lead to the emergence of an experience. Others have also taken a non-specialized vehicle-based approach (e.g., Mathis and Mozer, 1996; Zeki, 2001). Mathis and Mozer proposed that the contents of consciousness correspond to transiently stable states in an interconnected network of computational modules. These authors developed a model of a possible module that incorporated an attractor architecture, and felt that the development of a stable attractor was necessary for awareness. Again there was no attempt to explain how the development of attractors leads to a phenomenal outcome.

An important insight into how higher representational states could be generated came with the idea of representational re-description, where a system is able to reflect on its own internal states (Clark and Karmiloff-Smith, 1993). Theses ideas underpin proposals such as the “radical plasticity thesis” (Cleeremans, 2008) where a system is able to re-describe its own activities to itself. They are also key to the idea of metacognition requiring higher-order representations that emerge when the system observes its own internal states through having access to relevant lower-order knowledge (Pasquali et al., 2010). The focus of the current hypothesis is more on how these higher representational states can be generated within networks themselves.

The paper is highlighting the fact that at some point any hypothesis has to address the question of where the phenomenal component of consciousness arises from. What is it that causes an experience? The current work attempts to address this question directly, by looking at the way the information messages assigned by networks are changing during attractor behavior. It would argue that lower-order networks should be able to generate qualia if they are excited to attractor states, but so too could higher-order networks engaged in metacognitive activities. It is assumed that a large number of coincident attractors would result from the general brain-wide constraint satisfaction that many theories imply settles down at the moment of a conscious perception. It is very likely that the ability of any given specialized network to settle into an attractor state is going to be dependent on its interaction with other networks. The top-down focusing provided by re-entrant feedback is just one of these interactions. The interactions will inevitably lead to various transient activations that don’t enable networks to settle into attractors. The competition going on between networks would ultimately lead to a point where there is a set of winners that have activity sustained long enough for attractors to form and continue for few cycles. Each of these stable attractor states would provide qualia. The sum of all these qualia, as the global constraint is satisfied, would form the subjective conscious experience at that moment. So the theory is perfectly compatible with process theories of consciousness such as Global Workspace, and its competition between unconscious processors leading to winners accessing a global workspace. Therefore the proposed theory is just saying that no-matter what process is involved in the organizing of consciousness there has to be at some point a mechanism that causes the generation of phenomenal experience, and it is suggesting a mechanism that could generate it.

## Evidence for the Behavior Discussed

### Cortical network microanatomy

How well does the theory outlined above map onto the known microanatomy and dynamics of local networks in the cerebral cortex? The theory revolves around the establishment of repeated cycles of activity through cortical networks following local feedback, and for this activity to be under top-down re-entrant facilitation. For this to occur there are two key requirements.

- There needs to be large amounts of local feedback from pyramidal cells in the network back to the network again.
- There needs to be connections to layer 1 of the cortex to receive top-down re-entrant inputs, either from other areas in the cortical sensory hierarchy, or from the prefrontal cortex.

A number of local networks have substantial local feedback, particularly those involving layer 2/3 pyramidal cells (Thomson et al., 2002a). For direct access to the top-down inputs received by layer 1, the cells require apical dendrites that extend into this uppermost layer. Two networks stand out as satisfying these anatomical requirements. First of all networks in layer 2/3, where there are extensive local connections between layer 2/3 pyramids, and where the pyramidal cells have apical dendrites that climb to layer 1 (Thomson et al., 2002b). Secondly networks in layer 5, particularly those in the upper part of the layer with pyramidals in layer 5a. When the large pyramidal cells are close to each other in this layer they are particularly densely interconnected (Markram et al., 1997). These cells have a very robust climbing apical dendrite that forms a well-developed tuft in layer 1 (Thomson and Lamy, 2007). Pyramidal cells in layer 4 can have apical dendrites extending into layer 1 but the bulk of the cells in this layer are spiny stellate cells that have just local inputs. The layer 4 stellate cells do have high levels of local connectivity however (Lund, 1973). Layer 6 pyramidal cells have less extensive local feedback, and only the sparse claustrum-projecting cells have apical dendrites that can reach up to layer 1 (Katz, 1987). It is felt that the local connectivity requirements demanded by the theory outlined are well met by networks in layer 2/3 and layer 5a. For a comprehensive review of local circuit connectivity in the neocortex, see Thomson and Lamy (2007).

### Cortical network dynamics

It has been a key part of theoretical thinking since the 1980s that attractor behavior in local cortical networks underpins the development of concepts or percepts (Hopfield, 1982; Amit, 1989). However reliable evidence that these states are achieved has been hard to come by. This is partly because such states result from the behavior of quite large populations of neurons, and it is not straightforward analyzing the behavior of large numbers of cells to explore their population dynamics. Direct observation of population behavior has been made somewhat easier with the development of optical imaging techniques, particularly 2-photon calcium imaging (Wallace and Kerr, 2010). Although the indication of cell firing is indirect there is strong evidence that the calcium transients that are imaged closely reflect cell firing (Kerr et al., 2005). Using this technique Cossart et al. (2003) reported activity that closely resembled attractor behavior, interestingly primarily in layer 2/3 and layer 5 networks. Others have reported similar behaviors (e.g., Ikegaya et al., 2004). On the other hand Durstewitz and Deco (2008) have presented evidence that stable attractor states are often not achieved. They showed that quasi-stable states that are attractors in the making can contain useful information (Mazor and Laurent, 2005) and have suggested may be the primary manifestation of evolving percepts.

If information is being repeatedly cycled through local networks when activated by top-down feedback then you might expect to detect some oscillations in the local field potentials (LFPs) recorded in activated networks, and in scalp recordings of electric or magnetic fields. Crick and Koch (1990) suggested over 20 years ago that synchronous neural firing at the gamma frequency might be a neural correlate of visual awareness. Episodes of induced gamma activity that are not time-locked to the stimulus, and can be generated by anticipation and imagination as well as sensory input (Tallon-Baudry, 2003), are conjectured to be linked to top-down feedback, as part of the perception process (Fisch et al., 2009; Privman et al., 2010; Martinovic and Busch, 2011). This stimulus-linked gamma activity could reflect the cyclic feedback activity under re-entrant feedback that this paper predicts. It has long been reported that EEG gamma oscillations are associated with networks that are under attentional focus (Fries et al., 2001; Womelsdorf et al., 2006) with a marked increase in gamma power with attention. In monkey visual cortex this increase has been well documented in V4 (Taylor et al., 2005; Chalk et al., 2010). Chalk and colleagues have shown differential responses, with an enhancement in V4 and concomitant decrease in V1, as would be expected depending on the focus of attention. Gaillard et al. (2009) monitored intracranial potentials during conscious access and showed a marked increase in high-gamma power when access was achieved.

There is some evidence for a link between gamma frequency EEG responses and the establishment of conscious pain percepts. The typical short latency evoked slow EEG response has long been thought to reflect pain perception (e.g., Tzabazis et al., 2011). However with repetitive brief pain stimuli the amplitude of the slow response decreases with each repeat, despite a report of the same pain level by the subject (Iannetti et al., 2008). Therefore it is now concluded that the slow response is more likely reflecting attentional processes (Legrain et al., 2011). Brief bursts of gamma activity are also induced by brief pain stimuli, and correlate closely with the pain reported (Gross et al., 2007). The power of the induced gamma responses does not decrease with stimulus repetition, and these gamma responses are now concluded to more accurately reflect processes underlying pain perception (Zhang et al., 2012).

From modeling work, it would seem that oscillations in the gamma band are primarily driven by fast spiking interneurons (Wang and Buzsaki, 1996; Bartos et al., 2007). Clearly gamma activity as detected in EEGs and LFPs is likely to primarily reflect activity in pyramidal cells, because of their numerical predominance and microanatomy, but the dynamics of these local oscillations are probably controlled by the interneuron activity. Their activity could set the sampling rate for the network (Buzsaki and Chrobak, 1995), providing the time window for spatial pattern recognition.

### Predictions

There are a number of predictions that result from the theory outlined. Under conditions of top-down activation, such as during attentional focus, firing patterns of pyramidal cells in local networks should repeat over several cycles. This response should be more likely in layer 2/3 or 5a networks. This activity should stop if the attentional focus shifted, such as following presentation of a distractor. Such work could be done in awake animals. It would be useful if such work could be used to provide an EEG or MEG signature that could be explored in awake humans. Such a signature could be a gamma frequency response, perhaps with a particular time course and power envelope, perhaps with a particular spread of frequencies. To provide the link with qualia, subsequent work would need to be done on human subjects. If it were possible of course, the ideal signature would be obtained from humans, in parallel with detection of attractor behavior during surgical procedures.

If a signature of local attractor behavior could be obtained then it would predicted that it should correlate with the conscious report of the subject. For example if the subject were presented with a blue light stimulus it would be expected that the signature would be detected and localized to the V4 area. Both the amplitude or power of the response, and its duration, should reflect the subjects report. If a pair of brief sensory inputs were presented with reducing separation until the transient percepts merged, then the signature would also start as separate waveforms that merged at the same point as the percepts.

A number of experimental approaches have been used to explore correlations between neural activity and subjective responses (see Rees, 2007 for review). Activity relating to a stimulus can be recorded in the visual cortex during unconscious processing following techniques such as masking. It would be expected that such activity would not lead to the attractor signature. The signature should be detected during activity relating to illusory figures or during hallucinations. Activity relating to binocular rivalry should also only reflect the signature during the perceived part of the alternating cortical response. Detailed examinations of cortical activity during binocular rivalry can show responses in the suppressed area that are still sufficient for the experimenter to identify the stimulus, but that nevertheless remains phenomenally invisible to the subject (Rees, 2007). Such activity should still only generate the signature on the side perceived by the subject. Phosphenes resulting from transcranial magnetic stimulation should lead to the signature being generated.

## Conclusion

Both theoretical thinking and experimental work has shown the importance of re-entrant feedback on the emergence of conscious percepts in the sensory cortex. The paper has argued that one conclusion would be that conscious percepts result in some way from the local activity of the cortical networks receiving the top-down feedback. From an examination of the information processing capabilities of local networks it has been shown that interesting properties emerged if the local networks were encouraged to establish attractor dynamics. It was that shown that, as the network repeatedly receives and processes locally fedback information, the identity of the fedback information changes. Starting as a simple identification it evolves through a likeness, to an appearance, and to an experience. So the repeated recognition of its own representations could enable purely physical activities to have a phenomenological outcome. It is assumed that a necessary condition for this to occur is for the network to have top-down activation in order for the attractor behavior to be facilitated. There is some evidence that such behavior could occur, and there are a number of predictions of the theory that could be explored.

## Conflict of Interest Statement

The author declares that the research was conducted in the absence of any commercial or financial relationships that could be construed as a potential conflict of interest.
